# Similar CNV Neurodynamic Patterns between Sub- and Supra-Second Time Perception

**DOI:** 10.3390/brainsci11101362

**Published:** 2021-10-16

**Authors:** Mingming Zhang, Keye Zhang, Xing Zhou, Bin Zhan, Weiqi He, Wenbo Luo

**Affiliations:** 1 Research Center of Brain and Cognitive Neuroscience, Liaoning Normal University, Dalian 116029, China; zmm1001psy@lnnu.edu.cn (M.Z.); psyzhouxing@outlook.com (X.Z.); 2 Key Laboratory of Brain and Cognitive Neuroscience, Dalian 116029, China; 3 School of Social and Behavioral Sciences, Nanjing University, Nanjing 210023, China; zhangkeye@smail.nju.edu.cn; 4 State Key Laboratory of Brain and Cognitive Science, CAS Center for Excellence in Brain Science and Intelligence Technology, Institute of Psychology, Chinese Academy of Sciences, Beijing 100101, China; zhanb@psych.ac.cn; 5 Department of Psychology, University of Chinese Academy of Sciences, Beijing 100049, China

**Keywords:** temporal discrimination, sub-second time perception, supra-second time perception, contingent negative variation

## Abstract

In the field of time psychology, the functional significance of the contingent negative variation (CNV) component in time perception and whether the processing mechanisms of sub- and supra-second are similar or different still remain unclear. In the present study, event-related potential (ERP) technology and classical temporal discrimination tasks were used to explore the neurodynamic patterns of sub- and supra-second time perception. In Experiment 1, the standard interval (SI) was fixed at 500 ms, and the comparison interval (CI) ranged from 200 ms to 800 ms. In Experiment 2, the SI was fixed at 2000 ms, and the CI ranged from 1400 ms to 2600 ms. Participants were required to judge whether the CI was longer or shorter than the SI. The ERP results showed similar CNV activity patterns in the two experiments. Specifically, CNV amplitude would be more negative when the CI was longer or closer to the memorized SI. CNV peak latency increased significantly until the CI reached the memorized SI. We propose that CNV amplitude might reflect the process of temporal comparison, and CNV peak latency might represent the process of temporal decision-making. To our knowledge, it is the first ERP task explicitly testing the two temporal scales, sub- and supra-second timing, in one study. Taken together, the present study reveals a similar functional significance of CNV between sub- and supra-second time perception.

## 1. Introduction

Time perception is a fundamental part of the human experience. It is crucial for the survival of individual organisms and daily behaviors, including interpersonal communication [1,2,3]. Moreover, different time interval ranges are implicated in different types of perceptions and behaviors [4]. For example, music recognition and dancing are generally associated with millisecond perception [4,5], while individual decision making often takes several seconds or more time [4,6,7]. Many studies have investigated the topic of sub- and supra-second time perception by using diverse experimental paradigms and technologies. However, no consensus exists regarding whether the information processing mechanisms involved in sub- and supra-second time perception are similar or different.

Most studies posit that distinct processing mechanisms and neural systems are recruited to perceive the two types of intervals [4,8]. For example, in a direct comparison of sub- and supra-second timing in the human brain using functional imaging, Lewis and Miall [9] found that although many brain regions were involved in both sub- and supra-second durations, there were differences in activation patterns, suggesting that distinct components are involved in these two durations. Using a comprehensive, voxel-wise meta-analysis, Wiener et al. [10] found that sub-second timing tasks showed a higher propensity to recruit subcortical networks (e.g., the basal ganglia and cerebellum), whereas supra-second timing tasks were more likely to activate cortical structures (e.g., the supplementary motor area and prefrontal cortex). Hayashi et al. [4] provided neuroanatomical evidence that the bilateral anterior cerebellum was implicated in the sub-second condition (700 ms), and the inferior parietal cortex was associated with the supra-second condition (3500 ms).

However, other studies posit the view that sub- and supra-second time perceptions have a common mechanism [11]. Rammsayer and Ulrich [12] employed a dual-task approach and sensory interference paradigm to assess whether qualitatively different timing mechanisms were involved in the processing of 100 ms and 1000 ms. Their results argue against two qualitatively distinct timing mechanisms but are consistent with attention-based cognitive models of human timing. Recently, Rammsayer and Troche [13] applied confirmatory factor analysis to investigate the internal structure of interval timing performance in sub- and supra-second ranges. Their results argue against the validity of the distinct timing hypothesis. They also found that although a common timing mechanism model fitted the empirical data better than one based on the distinct timing hypothesis, the outcome of their confirmatory factor analyses supported the notion of two functionally related timing mechanisms underlying interval timing in sub- and supra-second ranges, respectively. Therefore, the mechanisms involved in sub- and supra-second time perceptions need to be explored more rigorously.

Event-related potential (ERP) technology has been used widely to measure the temporal features of individual cognition processing. Numerous ERP studies on time processing have found that the contingent negative variation (CNV), a slow cortical potential wave of negative polarity at fronto-central scalp locations, provides an on-line timing index. However, there is still disagreement about the cognitive function of the CNV component in time perception. Many studies have assumed that CNV amplitude indexed stimulus duration and varied with the length of time perception; that is, larger CNV amplitudes were observed in conditions with longer temporal stimulus and longer duration perception, reflecting the neural accumulator function during duration estimation [14,15,16,17,18]. However, some studies do not find these CNV amplitude results [19,20,21]. Additionally, the latency of the CNV peak seems to be related to the target duration even when the current temporal stimulus had a different duration. Specifically, several studies found that CNV peak latency developed during signal presentation, peaked around the target duration, and then declined when the current signal was longer than the target duration, providing a memory trace of the encoded target duration, which might be associated with the decision-making process [20,22,23,24,25,26,27]. In other words, the memory trace of the target duration might determine both the participant’s decision and the time course of the CNV [20].

Most of these ERP studies focus on short or long temporal interval processing. Only a few have directly explored CNV component patterns during sub- and supra-second time perception within one temporal scale [18,21]. For example, Zhang et al. [18] adopted a time bisection task to explore the difference between sub- and supra-second interval processing (400–1600 ms). Their results showed a significant hemispheric effect that CNV amplitude of the right hemisphere was significantly higher than that of the left hemisphere in the 400 ms condition, but not in the 1600 ms condition; a greater CNV amplitude was induced by 400 ms than 1600 ms, but CNV activity was not reported in the other five conditions (i.e., 600 ms, 800 ms, 1000 ms, 1200 ms, and 1400 ms). Ng et al. [21] employed a similar task (800–3200 ms) to explore how representations of anchor durations were used to make categorical decisions and found that CNV increased in amplitude up to the value of the short anchor, remained at a constant level until about the geometric mean of the short and long anchors, and then began to resolve. It is worth noting that these two studies both explored the ERP patterns of sub- and supra-second intervals in one experimental procedure. If the two intervals were separated for exploration, would we obtain similar or different results from previous studies? The present study, therefore, aimed to perform two experiments to investigate the functional meaning of CNV and similarities or differences between the two types of time perception.

In addition, experimental paradigms commonly used in previous ERP studies include temporal bisection, temporal generalization, and temporal discrimination tasks. In the generalization task, participants have to determine whether a presented duration is the same as or different from the standard interval (SI) learned at the beginning of the experiment. In the bisection task, participants have to determine whether a presented duration is closer than one of two previously learned short or long SIs. In these two paradigms, the SI is presented at the beginning of each block, and there may be a recruitment of long-term memory so that there is an additional cognitive demand for participants [28]. Therefore, the current study adopted the temporal discrimination task in which each trial started with the SI. This task is easier for participants to understand and execute.

The current study aimed to use the visual temporal discrimination task to explore the temporal dynamics of sub- and supra-second time perception, as well as the similarities or differences between the two types of duration processing mechanisms. For this purpose, we adopted a SI of 500 ms for the sub-second task (Experiment 1) and 2000 ms for the supra-second task (Experiment 2) [29,30]. On the basis of previous ERP studies, we hypothesized that, regardless of its sub- or supra-second time perception, CNV amplitude would be significantly more negative as the length of the current comparison interval (CI) increased, and that CNV peak latency would increase significantly and then stop when the current duration reached the memorized SI. It should also be noted that because of a small but robust difference in duration judgments between males and females [31,32,33], only male participants were recruited for the present study.

## 2. Experiment 1: ERP Study of Sub-Second Temporal Discrimination

### 2.1. Methods

#### 2.1.1. Participants

As paid volunteers, 23 male participants were recruited from the community around Liaoning Normal University. All participants were right-handed and had normal or corrected-to-normal vision. They did not self-report any severe physical diseases or mental disorders. They signed the written informed consent before participating in this experiment. Two males were excluded because their half ERP trials in most conditions were cut off. Thus, 21 males were finally analyzed (aged 34.90 ± 4.55 years). The study was approved by the Ethics Committee of Liaoning Normal University in accordance with the Declaration of Helsinki (1991).

#### 2.1.2. Procedure

A classical temporal discrimination task was used, and the procedure was established with E-Prime 2.0 software (Psychology Software Tools, Inc., Sharpsburg, PA, USA). Participants sat comfortably on a chair, 70 cm away from a 19-inch monitor screen (1440 × 900 pixels, 60 Hz refresh rate) in a soft, soundproofed room. All stimuli and fixation were presented in a central location on the computer screen with a black background.

**Figure 1:**
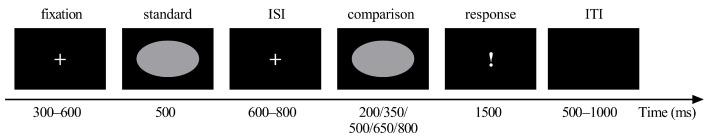
A depiction of the sub-second temporal discrimination task in Experiment 1. ISI: inter-stimulus interval. ITI: inter-trial interval.

At the beginning of each trial, a white fixation cross “+” was displayed for 300–600 ms. Subsequently, a ray ellipse (12 × 16 cm) appeared in the center of the screen for 500 ms (SI). After that, a white fixation cross “+” was presented between 600 ms and 800 ms (i.e., ISI). Referring to previous studies [24,34], a variable ISI was used to weaken participants’ expectation effect and keep the ERPs baseline stable. Then, participants were presented with a gray ellipse (12 × 16 cm) lasting 200 ms, 350 ms, 500 ms, 650ms, or 800 ms. When the CIs disappeared, a white exclamation mark “!” would appear. Participants were required to judge whether the second ellipse (CI) was longer or shorter than the first ellipse (SI) by pressing “F” or “J” on the keyboard, respectively. Responses with latencies less than 1500 ms were considered valid, and the ITI was displayed for 50–1000 ms (see Figure 1). The response key was counterbalanced across participants, and no feedback was provided. Besides, even though people generally cannot count or beat time properly during the very short intervals below one second, to avoid the potential influence of timing categories, in the instruction, all participants were told to view it passively and not to count or beat time during the whole procedure, if not, the results would be distorted [35]. All trials were presented in a randomized order in 10 blocks of 30 trials (i.e., 60 trials for each CI condition). Each block contained 6 repetitions of each CI condition. The participants took a full rest between the blocks.

To ensure that the participants had a good understanding of the task, a practice phase before the formal experiment would be conducted, in which each CI condition contained four trials, that is, 20 trials in total. Experiment 1 took approximately 30 min in total.

#### 2.1.3. Behavioral Data Analysis

The one-way repeated-measures analyses of variance (ANOVA) with five CIs was performed to test the mean proportion of “Long”. *p* values were corrected using the Greenhouse–Geisser method. The further pairwise comparisons were performed using Bonferroni correction. All statistical analyses were conducted using SPSS 26.0 for Windows.

#### 2.1.4. EEG Recording and Data Analysis

The EEG was recorded from 64 scalp sites using the electrodes mounted on an elastic cap (Brain Products, Munich, Germany) according to the standard 10–20 system, with a reference electrode of the left mastoid. The horizontal electrooculogram (EOG) was monitored from two electrodes situated 1 cm from the outer canthi of both eyes. The vertical EOGs were monitored from two electrodes positioned 1 cm above and below the right eye. The EEG and EOG signals were filtered with a band-pass of 0.01–100 Hz and all channels were recorded with a sampling rate of 500 Hz. For all electrodes, impedances were kept below 5 kΩ.

Analyzer 2.0 software (Brain Products, Munich, Germany) and EEGLAB [36] for MATLAB (MathWorks, MA, USA) were used to analyze the offline EEG data. The bilateral mastoid process was used as an off-line reference. Major artifacts, such as ocular movement, eyeblinks, and muscle-related potentials, were corrected with independent component analysis. The data were then digitally filtered at 0.01–30 Hz and segmented from 200 ms before to 1200 ms after the second ellipse (i.e., CI) onset. After baseline correction (−200 to 0 ms), trials with amplitudes exceeding ± 70 μV at any electrode were excluded from the average in order to eliminate the contamination of larger artifacts. This resulted in available trials in each CI condition for further analysis (M ± SE; 200 ms: 56.67 ± 0.91, 350 ms: 57.24 ± 0.97, 500 ms: 57.95 ± 0.86, 650 ms: 57.43 ± 0.68, 800 ms: 57.33 ± 0.93).

On the basis of previous studies [18,24,37] and the present total average waveform, CNV components were observed during the presentation of the CIs, and we selected a fronto-central electrode cluster (i.e., Fz, F3, F4, FCz, FC3, FC4, Cz, C3, and C4) for further analyses. Based on a visual inspection of the averaged waveforms, we used prior methods to assess the peak latency and mean amplitude of CNV for each condition and each participant [22,24]. Specifically, we calculated mean CNV amplitudes over 100-ms sliding time windows with a midpoint ranging from 300 ms to 1000 ms, and the peak latency was determined by the midpoint of the sliding window with the maximum amplitude. In addition, the average amplitudes from 300 ms to peak latency under each condition were calculated as the mean CNV amplitude.

Two-way repeated-measures ANOVA with 5 (CI duration: 200/350/500/650/800 ms) × 3 (hemisphere: left/middle/right) and three-way repeated-measures ANOVA with 2 (category: short/long) × 2 (duration difference from SI: smaller/larger) × 3 (hemisphere: left/middle/right) were performed on CNV peak latency and CNV amplitude. *p* values were corrected by using the Greenhouse–Geisser correction. Effect sizes were reported as partial eta squared ($\eta_{p}^{2}$). All statistical analyses were conducted using SPSS 26.0 for Windows.

### 2.2. Results

#### 2.2.1. Behavioral Results

**Figure 2:**
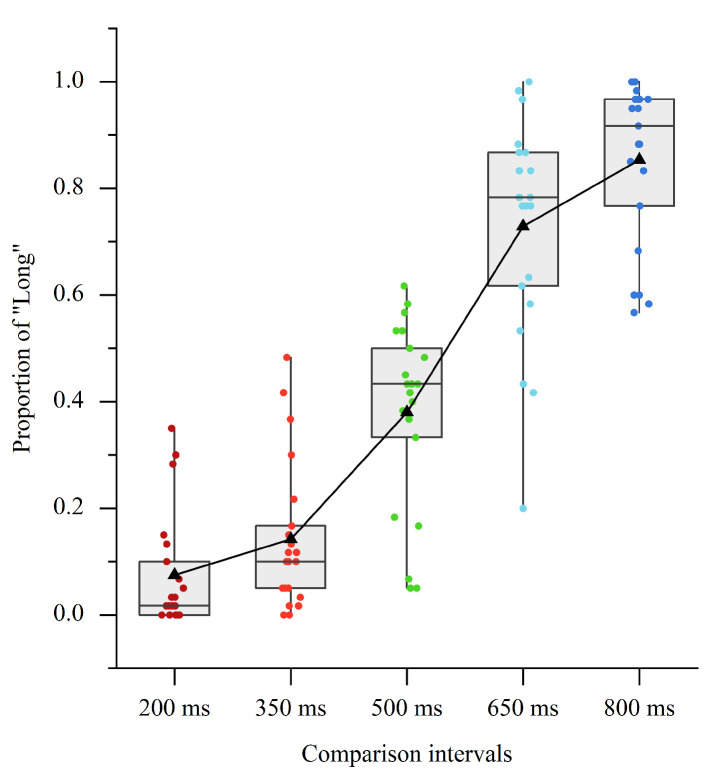
Boxplots of proportion of “Long” response in Experiment 1. The black triangles indicate mean values.

The main effect of CI condition was significant [F (4, 80) = 123.21, *p* < 0.001, $\eta_{p}^{2}$ = 0.86]. The further test revealed significant pairwise differences in the proportion of “Long” among the five CIs (M ± SE; 200 ms: 0.075 ± 0.024; 350 ms: 0.142 ± 0.030; 500 ms: 0.380 ± 0.038; 650 ms: 0.729 ± 0.045; 800 ms: 0.853 ± 0.034; *ps* ≤ 0.015; see Figure 2), suggesting that the participants could make clear discrimination in the current sub-second time perception.

#### 2.2.2. ERP Results

##### CNV Amplitude

**Figure 3:**
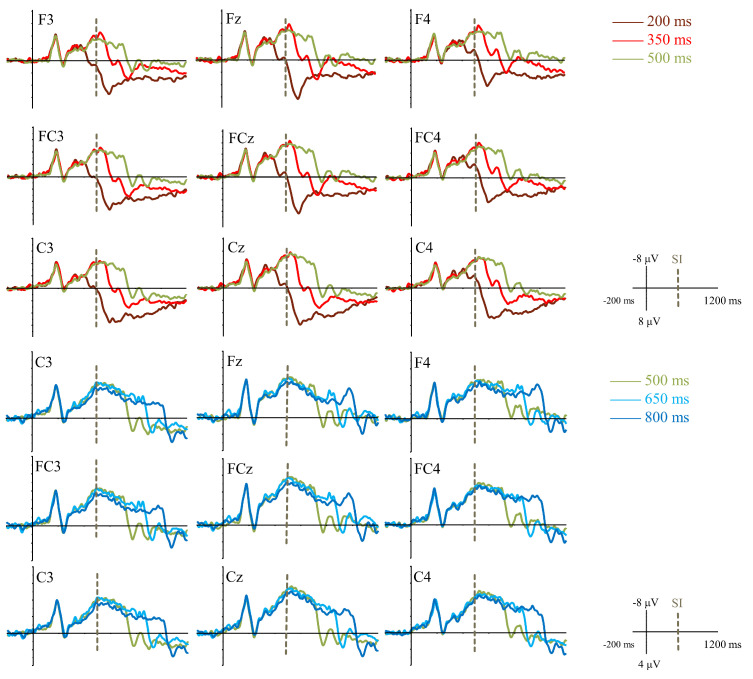
Grand average ERPs induced by different comparison intervals in Experiment 1.

Grand average ERPs induced by different CIs are shown in Figure 3. Two-way repeated-measures ANOVA analysis revealed a significant main effect of CI condition [F (4, 80) = 3.59, *p* = 0.016, $\eta_{p}^{2}$ = 0.15]. Further pairwise comparisons showed that CNV negative wave induced by 200 ms condition (*M* ± *SE*; −2.64 ± 0.42 μV) was the smallest and significantly smaller than the other four CI conditions (350 ms: −3.41 ± 0.38 μV; 500 ms: −3.43 ± 0.35 μV; 650 ms: −3.47 ± 0.34 μV; 800 ms: −3.24 ± 0.44 μV; *p*s < 0.05). Moreover, the differences among CNV amplitudes induced by the other four CI conditions were not significant (*p*s > 0.05). In addition, the main effect of hemisphere was also significant [F (2, 40) = 19.18, *p* < 0.001, $\eta_{p}^{2}$ = 0.49]. CNV amplitude in the left brain area (−2.61 ± 0.33 μV) was smaller than that in right (−3.31 ± 0.31 μV; *p* = 0.001) and middle brain areas (−3.80 ± 0.46 μV; *p* < 0.001). CNV amplitude in the right brain area was smaller than that in the middle brain area (*p* < 0.05). The interactive effect of CI condition and hemisphere was not significant [F (8, 160) = 1.00, *p* > 0.05, $\eta_{p}^{2}$ = 0.05].

Three-way repeated-measures ANOVA analysis showed a significant main effect of category [F (1, 20) = 5.20, *p* < 0.05, $\eta_{p}^{2}$ = 0.21]. Compared with the CI condition shorter than SI (*M* ± *SE*; −3.03 ± 0.37 μV), CNV amplitude induced by the CI condition longer than SI (−3.36 ± 0.37 μV) was more negative. The main effect of duration difference from SI was significant [F (1, 20) = 6.39, *p* < 0.05, $\eta_{p}^{2}$ = 0.24]. The CI condition with a smaller duration difference from SI (−3.44 ± 0.34 μV) elicited a more negative CNV than the CI condition with a larger duration difference from SI (−2.94 ± 0.42 μV). Furthermore, there was a significant main effect of hemisphere [F (2, 40) = 17.39, *p* < 0.001, $\eta_{p}^{2}$ = 0.47]. CNV amplitude in the left brain area (−2.57 ± 0.35 μV) was smaller than that in right (−3.26 ± 0.31 μV; *p* < 0.01) and middle brain areas (−3.74 ± 0.48 μV; *p* < 0.001), and CNV amplitude in right brain area was smaller than that in middle brain area (*p* = 0.05). No significant interactive effects were found (*p*s > 0.05).

##### CNV Latency

Two-way repeated-measures ANOVA analysis showed a significant main effect of CI condition [F (4, 80) = 15.22, *p* < 0.001, $\eta_{p}^{2}$ = 0.43]. Specifically, CNV peak latency induced by 200 ms (*M* ± *SE*; 426.82 ± 21.22 ms) was significantly shorter than the other four CI conditions (350 ms: 503.48 ± 9.16 ms; 500 ms: 549.02 ± 16.16 ms; 650 ms: 570.83 ± 19.31 ms; 800 ms: 618.39 ± 37.98 ms; *p*s < 0.001). CNV peak latency induced by 350 ms was significantly shorter than that induced by 500 ms, 650 ms, and 800 ms (*p*s < 0.01). Additionally, the differences in CNV peak latency among 500 ms, 650 ms, and 800 ms were not significant (*p*s > 0.05). Furthermore, the main effect of hemisphere was significant [F (2, 40) = 7.92, *p* = 0.001, $\eta_{p}^{2}$ = 0.28]. CNV peak latency in right brain area (559.61 ± 16.41 ms) was significantly longer than CNV amplitude in left (525.16 ± 5.43 ms; *p* < 0.05) and middle brain areas (516.35 ± 18.93 ms; *p* < 0.001). CNV peak latency in the left brain area did not differ from that in the middle brain area (*p* > 0.05). Their interactive effect was not significant [F (8, 160) = 1.10, *p* = 0.365, $\eta_{p}^{2}$ = 0.05].

Three-way repeated-measures ANOVA analysis showed a significant main effect of category [F (1, 20) = 36.17, *p* < 0.001, $\eta_{p}^{2}$ = 0.64]. Compared with the CI condition shorter than SI (*M* ± *SE*; 465.15 ± 13.37 ms), CNV peak latency induced by the CI condition longer than SI (594.61 ± 25.56 ms) was longer. There was a significant main effect of hemisphere [F (2, 40) = 6.68, *p* < 0.01, $\eta_{p}^{2}$ = 0.25]. CNV peak latency in right brain area (556.60 ± 19.22 ms) was significantly longer than that in left (522.59 ± 17.10 ms; *p* < 0.05) and middle brain areas (510.45 ± 20.26 ms; *p* < 0.01). The difference between the left and middle brain area was not significant (*p* > 0.05). Furthermore, the interactive effect of category and duration difference from SI was significant [F (1, 20) = 14.13, *p* = 0.001, $\eta_{p}^{2}$ = 0.41]. The further simple effect test results showed that, only in the CI condition shorter than SI, CNV peak latency induced by the CI condition with a smaller duration difference from SI (503.48 ± 9.16 ms) was significantly longer than the CI condition with a larger duration difference from SI (426.82 ± 21.22 ms; *p* < 0.01). The main effect of duration difference from SI and other interactive effect was not significant (*p*s > 0.05).

## 3. Experiment 2: ERP Study of Supra-Second Time Discrimination

### 3.1. Methods

#### 3.1.1. Participants

As paid volunteers, 23 males (aged 35.35 ± 4.48 years) were recruited from the community around Liaoning Normal University. Two of them were left-handed. All participants had normal or corrected-to-normal vision. They did not self-report any severe physical diseases or mental disorders. They signed the written informed consent before participating in this experiment. The study was approved by the Ethics Committee of Liaoning Normal University in accordance with the Declaration of Helsinki (1991).

#### 3.1.2. Procedure

**Figure 4:**
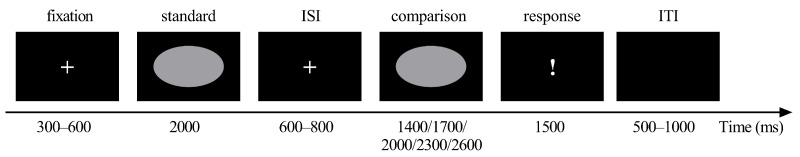
A depiction of the supra-second temporal discrimination task in Experiment 2. ISI: inter-stimulus interval; ITI: inter-trial interval.

The procedure of Experiment 2 was similar to that of Experiment 1 (see Figure 4). Experiment 2 used a temporal discrimination task, but five supra-second intervals served as CIs. Specifically, the durations of fixation cross “+”, exclamation mark “!”, and the task remained unchanged; the first gray ellipse (SI) lasted 2000 ms, and the second gray ellipse (CI) lasted 1400 ms, 1700 ms, 2000 ms, 2300 ms, or 2600 ms. For Experiment 1, the response key was counterbalanced across participants. No feedback was provided. All trials were presented in a randomized order in ten blocks of 30 trials, with each block containing six repetitions of each CI condition. Participants took a full rest between the blocks. Furthermore, participants carried out the practice experiment before the formal experiment. There was a total of 20 trials in the practice phase, with each CI condition containing four trials. Experiment 2 took approximately 45 min in total.

#### 3.1.3. Behavioral Data Analysis

The one-way repeated-measures ANOVA with five CIs was performed to test the mean proportion of “Long”. *p* values were corrected using the Greenhouse–Geisser method. The further pairwise multiple comparisons were performed using Bonferroni correction. All statistical analyses were conducted using SPSS 26.0 for Windows.

#### 3.1.4. EEG Recording and Data Analysis

EEG recording and preprocessing steps were similar to that in Experiment 1. This resulted in available trials in each CI condition for further analysis (*M* ± *SE*; 1400 ms: 47.30 ± 1.19; 1700 ms: 46.48 ± 1.67; 2000 ms: 47.83 ± 1.40; 2300 ms: 48.83 ± 1.30; 2600 ms: 49.00 ± 1.23). Additionally, we also assessed the peak latency and mean amplitude of CNV for each CI condition similar to Experiment 1. Mean CNV amplitudes over 100-ms sliding time windows with a midpoint ranging from 1000 ms to 3000 ms were calculated, and the peak latency was determined by the midpoint of the sliding window with the maximum amplitude. In addition, the average amplitudes from 1000 ms to peak latency under each condition were calculated as the mean CNV amplitude. Moreover, two-way repeated-measures ANOVA and three-way repeated-measures ANOVA were similar to that conducted in Experiment 1.

### 3.2. Results

#### 3.2.1. Behavioral Results

**Figure 5:**
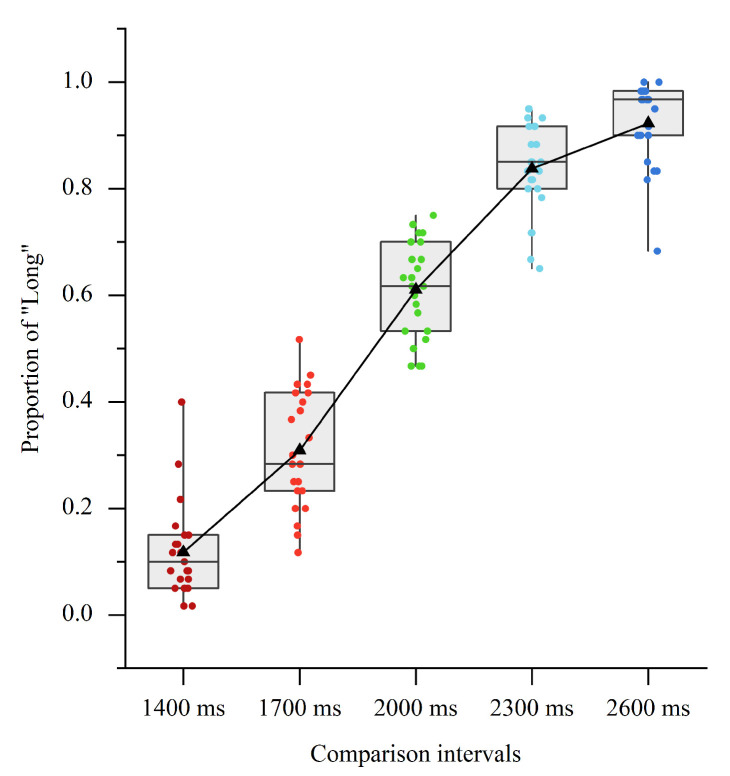
Boxplots of proportion of “Long” response in Experiment 2. The black triangles represent mean values.

The main effect of CI condition was significant [F (4, 88) = 366.20, *p* < 0.001, $\eta_{p}^{2}$ = 0.943]. The further test revealed significant pairwise differences in the proportion of “Long” among the five CIs (*M* ± *SE*; 200 ms: 0.117 ± 0.018; 350 ms: 0.309 ± 0.023; 500 ms: 0.610 ± 0.019; 650 ms: 0.838 ± 0.018; 800 ms: 0.922 ± 0.016; *p*s < 0.001; see Figure 5), suggesting the participants could make clear discrimination in the current supra-second time perception.

#### 3.2.2. ERP Results

##### CNV Amplitude

**Figure 6:**
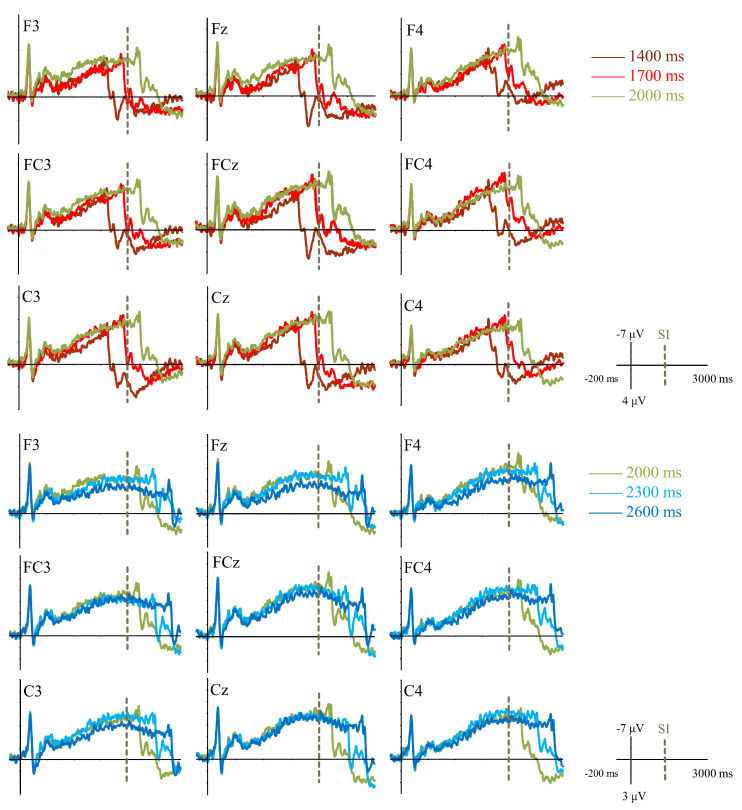
Grand average ERPs induced by different comparison intervals in Experiment 2.

Grand average ERPs induced by different CIs are shown in Figure 6. Two-way repeated-measures ANOVA analysis showed a significant main effect of CI condition [F (4, 88) = 3.89, *p* < 0.01, $\eta_{p}^{2}$ = 0.15]. CNV induced by 1400 ms (*M* ± *SE*; −2.15 ± 0.25 μV) was the smallest and was significantly smaller than the other four CI conditions (1700 ms: −2.91 ± 0.35 μV; 2000 ms: −3.32 ± 0.27 μV; 2300 ms: −3.34 ± 0.39 μV; 2600 ms: −2.88 ± 0.40 μV; *p*s < 0.05). Moreover, the differences among CNV amplitudes induced by the other four CIs were not significant (*p*s > 0.05). The main effect of hemisphere and interactive effect of CI condition and hemisphere were not significant (*p*s > 0.05).

Three-way repeated-measures ANOVA analysis showed a significant main effect of category [F (1, 22) = 4.51, *p* < 0.05, $\eta_{p}^{2}$ = 0.17]. Compared with the CI condition shorter than SI (*M* ± *SE*; −2.53 ± 0.25 μV), CNV amplitude induced by the CI condition longer than SI (−3.11 ± 0.35 μV) was more negative. Moreover, the main effect of duration difference from SI was significant [F (1, 22) = 5.33, *p* < 0.05, $\eta_{p}^{2}$ = 0.20]. The CI condition with a smaller duration difference from SI (−3.13 ± 0.32 μV) elicited a more negative CNV than the CI condition with a larger duration difference from SI (−2.51 ± 0.29 μV). The main effect of hemisphere and interactive effect of CI condition and hemisphere were not significant (*p*s > 0.05).

##### CNV Latency

Two-way repeated-measures ANOVA analysis showed a significant main effect of CI condition [F (4, 88) = 10.13, *p* < 0.001, $\eta_{p}^{2}$ = 0.32]. CNV peak latency induced by 1400 ms (*M* ± *SE*; 1400 ms: 1735.55 ± 56.67 ms) was significantly shorter than the other four CI conditions (1700 ms: 1830.55 ± 55.78 ms; 2000 ms: 1963.34 ± 59.75 ms; 2300 ms: 2050.37 ± 75.66 ms; 2600 ms: 2094.52 ± 62.53 ms; *p*s < 0.05). CNV peak latency induced by 1700 ms was significantly shorter than CNV peak latency induced by 2000 ms, 2300 ms, or 2600 ms (*p*s < 0.05). Additionally, the differences among CNV peak latency induced by 2000 ms, 2300 ms, and 2600 ms were not significant (*p*s > 0.05). Furthermore, the main effect of hemisphere was significant [F (2, 44) = 3.54, *p* = 0.037, $\eta_{p}^{2}$ = 0.14]. CNV peak latency in right brain area (2019.34 ± 51.39 ms) was significantly longer than that in middle brain area (1876.75 ± 56.57 ms; *p* < 0.05) and was not significantly longer than that in the left brain area (1908.51 ± 60.78 ms; *p* > 0.05). The difference between the left and middle brain areas was not significant (*p* > 0.05). The interactive effect of CI condition and hemisphere was not significant [F (8, 176) = 0.85, *p* = 0.52, $\eta_{p}^{2}$ = 0.04].

Three-way repeated-measures ANOVA analysis showed a significant main effect of category [F (1, 22) = 21.71, *p* < 0.001, $\eta_{p}^{2}$ = 0.50]. Compared with the CI condition shorter than SI (*M* ± *SE*; 1783.05 ± 52.03 ms), CNV peak latency induced by the CI condition longer than SI (2072.44 ± 61.13 ms) was longer. There was a significant main effect of hemisphere [F (2, 44) = 3.70, *p* < 0.05, $\eta_{p}^{2}$ = 0.14]. CNV peak latency in the right brain area (2012.31 ± 54.05 ms) was significantly longer than that in the middle brain area (1858.45 ± 59.11 ms; *p* < 0.05) and longer than that in the left brain area (1912.48 ± 60.45 ms; *p* > 0.05). The difference between the right and middle brain area was not significant (*p* > 0.05). Moreover, the interactive effect of category and duration difference from SI was significant [F (1,22) = 6.34, *p* < 0.05, $\eta_{p}^{2}$ = 0.22]. The further simple effect test results showed that, only in the CI condition shorter than SI, CNV peak latency induced by the CI condition with a smaller duration difference from SI (1830.55 ± 55.78 ms) was significantly longer than that in the CI condition with a larger duration difference from SI (1735.55 ± 56.67 ms; *p* < 0.05). The main effect of duration difference from SI and other interactive effects was not significant (*p*s > 0.05).

## 4. Discussion

The present study aimed to investigate the neurodynamics of sub- and supra-second time perception using the visual temporal discrimination paradigm, as well as similarities between the two types of duration processing mechanisms. We found that CNV component activity patterns of sub- and supra-second were largely similar.

Interestingly, some studies have argued that CNV amplitude varies with stimulus duration and length of time perception. Usually, the longer the temporal stimulus, the longer the time perception, and the larger CNV amplitude [14,15,16,18]. However, the ERP results in neither Experiment 1 nor Experiment 2 revealed this profile. Specifically, although CNV amplitude induced by the “longer CI condition” was indeed more negative than the “shorter CI condition”, the results of the main effect of the CI condition showed that CNV amplitude was not more negative with the increase in comparison during the stimulus. In other words, CNV amplitude does not directly reflect the unfolding of time [19]. Some previous studies do not find an association between CNV amplitude and perceived duration. For example, Macar and Vidal [20] used a temporal generalization task with a 2-s SI, and the analysis as a function of signal duration revealed no differences in CNV amplitude. Kononowicz and van Rijn [19] adopted the same duration reproduction paradigm used by Macar et al. [15] and did not find any related effects on CNV amplitude, even using more powerful linear-mixed effect analyses. Furthermore, the main effect of duration difference from SI suggests that the smaller the temporal difference in interval length, the more difficult the processing, and the more negative the amplitude of CNV. Therefore, we consider that CNV amplitude do not conform to the so-called “pacemaker-accumulator hypothesis” [17,38], and it is more likely to reflect the process of comparing the current CI with the memorized SI. The greater the attention resource is paid to the interval stimulus, the greater CNV amplitude [26]. Specifically, when the current CI is longer, and the difference between its interval length and the memorized SI is small, participants need attentional effort to complete the task accurately, so the CNV amplitude would also be larger and more negative. Additionally, increased negativity of CNV might also represent structured activity or increased mutual information between the processing of CI and SI. Thus, it could indicate that CI is closer to SI in working memory, suggested by increased mutual information.

Both Experiments 1 and 2 showed that CNV peak latencies induced by the three longer CIs (500/650/800 ms and 2000/2300/2600 ms, respectively) were not significantly different. These results are in line with the study by Kononowicz and van Rijn [34] who found that CNV peak latency increased significantly until the current CI reached the memorized SI. This also supports the viewpoint that CNV peak latency is an index of decision making [20,26]. Specifically, in temporal paradigms, the decision is based on a comparison between a current elapsing duration and a memorized target [20]. When the CI is shorter than the SI, participants could make a judgment and a decision at the end of the CI in their minds. Therefore, CNV peak latencies induced by the two shorter CI conditions (200/350 ms, 1400/1700 ms) are significantly shorter than the “CI condition equal to or longer than the SI”. However, this is not the case when the CI is longer than the SI. Rather than peaking at the end of the current CI, CNV peaks earlier. That is, when the interval length of the presented CI reaches the SI in memory, participants could produce a mental distinction between the two durations before the end of the current temporal stimulus presentation and then make a decision. Therefore, in these instances, CNV peak latency corresponds to the end of the comparison process and would not be significantly extended with an increase in the CI. Once the participant’s decision is taken, the last portion of the current duration will not have to be timed.

An alternative explanation of the similarity of CNV component patterns between sub- and supra-second time perception in the present study might relate to some theories of the psychological present. Fraisse [39] points out that psychological present has an upper limit of 5 s and has an average value of 2 s to 3 s. Pöppel [40,41] also proposes a hierarchical model in which time perception is controlled by two separate processing mechanisms in the brain. The first system is a high-frequency system with a period of 20–60 ms and an average of 30 ms; it is the primary integrated unit of human processing information. The second system is a low-frequency system that processes a series of events within a few seconds (2–3 s), integrating sequential events into units of perception and motion; it is the basis of the perceived present. A large number of studies are based on these views, revealing that there are different mechanisms for time processing below and above 3 s [42]. Specifically, it is not regulated by cognitive resources (attention and working memory) below 3 s and has cross-channel non-specificity. On the contrary, temporal processing above 3 s is regulated by cognitive resources and has channel specificity. These findings have been observed in the research using behavior [43,44,45,46], ERP [47], brain imaging [9], and nontraumatic brain stimulation [48,49]. In the present study, the duration of time perception ranged from 200 ms to 2600 ms, and all conditions were below 3 s, which means that participants might be lying within the 3 s interval of present moment awareness, resulting in similar CNV neurodynamics in Experiments 1 and 2. Therefore, it is worth exploring the CNV patterns of longer intervals (above 3 s) in our future research.

Additionally, we analyzed the hemispheric effect of the CNV component; the results showed that there were different hemispheric patterns between the two experiments. Regarding CNV amplitude, we found that there was a significant hemispheric effect in the sub-second condition (i.e., higher CNV amplitude in the right hemisphere than in the left hemisphere), but not in the supra-second condition. This finding is consistent with that of Zhang et al. [18]. As for CNV peak latency, we also found a significant hemispheric effect. CNV peak latency of the right hemisphere was significantly longer than that of the left hemisphere in the sub-second condition, but not in the supra-second condition, which is consistent with the study of Pfeuty et al. [22]. The different hemispheric patterns between sub- and supra-second time perception may be caused by the different roles of the left and right frontal lobes in information processing. Some studies have shown that the right frontal lobe played an important role in time perception, while the left frontal lobe was more likely to be involved in general attention and memory processes [50]. When the time perception task becomes more difficult and requires more attention resources, activation of the left hemisphere increases accordingly [18]. Consequently, the difference between the left and right hemisphere in the supra-second experiment would be reduced.

Several limitations of this study should be mentioned. First, the participants were homogenous. The results of our study should be verified among women. Second, we only adopted the temporal discrimination task to investigate the participants’ performance of time perception. Further studies should investigate the differences or similarities between sub- and supra-second time perception using different paradigms. Third, supra-second time perception in Experiment 2 ranged from 1.4 s to 2.6 s. Röhricht et al. [51] employed the classic S1–S2 paradigm task, an implicit timing task, with filled intervals of 2.5 s, 5 s, 7.5 s, and 10 s to investigate the maximum duration during which cortical CNV can be maintained. They found that intervals between 7.5 s and 10 s represented the upper boundary of the late CNV activation, and the continuously negative waveform of CNV ended at a point between 5 s and 7.5 s. Therefore, longer intervals (2.5–10 s) might be relevant to consciousness occurrence or information integration [52,53] and worth being included in the future research.

## 5. Conclusions

The present study used the ERP technique to investigate and compare the temporal dynamic characteristics of sub- and supra-second time perception. The findings showed that CNV component activity patterns of sub- and supra-second time perception were largely similar. CNV amplitude was more likely to reflect the process of comparing the current comparison interval with the memorized standard interval, and CNV peak latency might be an index of temporal decision-making.

## Data Availability

The data are available from the corresponding author upon reasonable requests.
